# Screening dataset of food components that enhance transcriptional activity of PGC1-beta

**DOI:** 10.1016/j.dib.2019.103814

**Published:** 2019-03-06

**Authors:** Shiho Nakai, Ran Uchitomi, Rintaro Matsuda, Takumi Onishi, Yukino Hatazawa, Yasutomi Kamei

**Affiliations:** Graduate School of Life and Environmental Sciences, Kyoto Prefectural University, Kyoto, Japan

**Keywords:** Screening, Reporter assay, Transcriptional activity

## Abstract

PGC-1β is a transcriptional co-activator of nuclear receptors, which acts to increase energy expenditure. PGC-1β fused to GAL4 DNA-binding domain transfected in HEK293T cells showed a reporter luciferase activity. We screened food-derived and natural compounds using a reporter assay system to measure the transcriptional activity of PGC-1β.

We found that soy-derived isoflavones, genistein and daidzein, and several resveratrols activated PGC-1β, see “Genistein, daidzein, and resveratrols stimulate PGC-1β-mediated gene expression” [1]. The list of 166 compounds and their reporter activity is shown here.

**Table:** Specifications table

Subject area	*Biology*
More specific subject area	*Food science*
Type of data	*Table*
How data was acquired	*Luciferase reporter assay, using Promega, GloMax Navigator System GM2010*
Data format	*Analyzed*
Experimental factors	*Cells, treated with food compounds, were lysed for luciferase assay.*
Experimental features	*We used PGC-1β fused with a GAL4 DNA-binding domain, which allows the measurement of transcriptional activation of PGC-1β in the presence of various compounds in the culture medium.*
Data source location	*Kyoto, Japan*
Data accessibility	*Contained within this article*
Related research article	[1]*R. Uchitomi, S. Nakai, R. Matsuda, T. Onishi, S. Miura, Y. Hatazawa, Y. Kamei. Genistein, daidzein, and resveratrols stimulate PGC-1β-mediated gene expression.****Biochemistry and Biophysics Reports****17:51-55, 2019*[1]

**Table:** 

**Value of the data** • The data could be used by researches, for example, in the food sciences, to evaluate food-derived and natural compounds as activators of PGC-1β, a transcriptional regulator that can enhance energy expenditure-related gene. • We made a fusion protein of PGC-1β with GAL4 DNA-binding domain, and established a system for screening PGC-1β-transcriptional activators. The system will be a practical example of screening system. • As in vivo activation of PGC-1β increases energy expenditure, PGC-1β-transcriptional activators could form the basis for anti-obesity dietary supplements.

## 1 Data

Food components and their reporter activity values as PGC-1β-transcriptional activators are listed. Chemical Names and Relative luc values are shown. The data from luciferase values in the presence of vehicle alone were set at 100. Data are expressed as mean ± SE (N = 3). P value < 0.05 was considered significant. ***P < 0.001, **P < 0.01, *P < 0.05 compared with the samples from in the presence of vehicle alone. Compounds that significantly increased luc activity were Baicalin, Caffeic Acid, Chrysin, Daidzein, 5, 7-Dimethoxyflavone, (-)-Epicatechin, Genistein, Homogentisic acid, (+/−)-Lavandulol, Lupeol, Luteolin, Quercetin, Resveratrol, trans-Oxyresveratrol, trans-Piceatannol, and trans-Pterostilbene. Compounds that significantly decreased luc activity were Daunorubicin hydrochloride, Magnolol, and trans-Ferulic acid (see Table 1).

**Table 1:** List of food-derived and natural compounds and their values of reporter activity as PGC-1β-transcriptional activators. Vehicle alone serves as the reference value (set as 100).

No.	Chemical Name	Relative Luc activity (%）		P value	
1	Abietate	113 ± 9	0.312		
2	Acacetin	106 ± 28	0.850		
3	Aconitine	120 ± 10	0.454		
4	Allicin	98 ± 9	0.862		
5	Allyl Disulfide < Diallyl Disulfide>	117 ± 15	0.416		
6	alpha-Mangostin	98 ± 31	0.960		
7	alpha-Santonin	93 ± 25	0.834		
8	alpha-Terpineol	99 ± 7	0.899		
9	Apigenin	205 ± 64	0.194		
10	Arbutin	99 ± 4	0.805		
11	(-)-Arctigenin	104 ± 9	0.884		
12	Arctiin	95 ± 8	0.619		
13	Astragaloside	138 ± 9	0.182		
14	Aucubin	111 ± 14	0.495		
15	Baicalin	135 ± 9	0.023		*
16	Barbaloin	125 ± 20	0.440		
17	Benzoic acid	108 ± 6	0.326		
18	Berberine Chloride	76 ± 8	0.057		
19	(-)-Bilobalide from Ginkgo biloba leaves	104 ± 33	0.921		
20	Borneol	119 ± 21	0.436		
21	Bornyl isovalerate	127 ± 14	0.242		
22	Caffeic Acid	136 ± 9	0.032		*
23	Capsaicin	168 ± 27	0.118		
24	(+/−)-Catechin hydrate	105 ± 14	0.850		
25	Chrysin	168 ± 18	0.020		*
26	Chrysophanol	107 ± 14	0.789		
27	cis-4-Hydroxycinnamic acid	87 ± 22	0.633		
28	Citrinin	98 ± 9	0.933		
29	Colchicine	200 ± 34	0.066		
30	Corosolic acid	105 ± 7	0.612		
31	4-Coumaric Acid	114 ± 11	0.313		
32	Cucurbitacin B	144 ± 55	0.494		
33	Curcumin 1 (Curcumin)	162 ± 16	0.084		
34	Curcumin 2	152 ± 26	0.197		
35	Curcumin 3	95 ± 18	0.878		
36	Daidzein	204 ± 17	0.007		**
37	Daunorubicin hydrochloride	53 ± 7	0.025		*
38	Dihydrocapsaicin	108 ± 12	0.565		
39	Dihydromyricetin	126 ± 17	0.388		
40	5,7-Dihydroxy-3-(4-hydroxy-phenyl)-chromen-4-one	136 ± 35	0.418		
41	3,3′-Diindolylmethane	84 ± 6	0.287		
42	5, 7-Dimethoxyflavone	160 ± 10	0.006		**
43	Diosgenin	118 ± 15	0.435		
44	Diosmetin	157 ± 9	0.065		
45	Diosmin	125 ± 20	0.433		
46	dl-Tetrahydroberberine (dl-Canadine)	113 ± 10	0.499		
47	Echinacoside	113 ± 7	0.588		
48	(-)-Epicatechin	174 ± 13	0.042		*
49	(-)-Epicatechin gallate	86 ± 21	0.591		
50	(-)-Epigallocatechin	123 ± 20	0.392		
51	(-)-Epigallocatechin gallate	154 ± 32	0.229		
52	Esculetin <Cichorigenin>	115 ± 11	0.312		
53	Evodiamine	121 ± 23	0.521		
54	Fucoxanthin	101 ± 13	0.966		
55	Fustin	102 ± 7	0.865		
56	Galangin	101 ± 3	0.937		
57	Gallic acid monohydrate	115 ± 15	0.583		
58	(-)-Gallocatechin gallate	79 ± 9	0.219		
59	Genistein	169 ± 21	0.034		*
60	Geraniol	121 ± 9	0.415		
61	Geranyl Acetate	123 ± 8	0.223		
62	Ginkgolic acid 15:0	124 ± 13	0.167		
63	Ginkgolide A	125 ± 4	0.171		
64	Ginkgolide B	172 ± 44	0.212		
65	Ginkgolide B	102 ± 13	0.935		
66	Ginkgolide C	141 ± 16	0.127		
67	Ginkgolide J	128 ± 11	0.204		
68	18β-Glycyrrhetinic acid	113 ± 16	0.489		
69	Glycyrrhizin (Glycyrrhizic acid)	139 ± 22	0.278		
70	Gomisin N	115 ± 6	0.157		
71	Gossypetin	120 ± 6	0.084		
72	Hesperetin	161 ± 20	0.105		
73	Hesperidin	119 ± 5	0.422		
74	(2S)-Hesperidin	117 ± 9	0.169		
75	Homogentisic acid	153 ± 8	0.031		*
76	Honokiol	109 ± 5	0.238		
77	3-(4-Hydroxy-3-methoxy-phenyl)-acrylic acid	128 ± 18	0.358		
78	3-Hydroxytyrosol	83 ± 10	0.307		
79	Icariin	113 ± 18	0.523		
80	Imperatorin	73 ± 7	0.024		*
81	Indole-3-carbino	122 ± 11	0.392		
82	Kaempferol	114 ± 21	0.658		
83	L-(+)-Ascorbic Acid	108 ± 4	0.333		
84	(+/−)-Lavandulol	120 ± 2	0.012		*
85	L-Deoxyalliin < S-Allyl-L-Cysteine>	131 ± 12	0.085		
86	Ligustilide	135 ± 19	0.222		
87	Limonene	132 ± 23	0.254		
88	Lupeol	137 ± 12	0.049		*
89	Luteolin	246 ± 40	0.032		*
90	Luteolin-7-O-Glucoside	121 ± 9	0.130		
91	Magnolol	40 ± 5	0.009		**
92	Mangiferin	104 ± 13	0.886		
93	Maslinic acid	105 ± 7	0.591		
94	Matrine	156 ± 31	0.211		
95	Melatonin	63 ± 9	0.063		
96	(-)-Menthone	90 ± 4	0.147		
97	(+)-Menthol	106 ± 21	0.820		
98	(+)-Menthone	135 ± 6	0.084		
99	Myricetin	159 ± 21	0.081		
100	Naringenin	131 ± 31	0.443		
101	Naringin	139 ± 12	0.176		
102	(2S)-Naringin	135 ± 17	0.238		
103	Naringin Hydrate	116 ± 8	0.385		
104	Neochlorogenic Acid	100 ± 5	0.988		
105	Neohesperidin	112 ± 13	0.430		
106	Nerolidol	108 ± 27	0.795		
107	Nordihydroguaiaretic acid	111 ± 27	0.728		
108	(+/−)-Octopamine hydrochloride	83 ± 10	0.328		
109	Oleanolic acid	118 ± 10	0.484		
110	Oroxylin A	134 ± 13	0.123		
111	Osthol	99 ± 10	0.902		
112	Osthole	104 ± 10	0.861		
113	Paclitaxel	98 ± 22	0.948		
114	Paeonol	118 ± 16	0.342		
115	Parthenolide	102 ± 3	0.922		
116	Pelargonidin	122 ± 12	0.298		
117	Pelargonidin chloride	130 ± 19	0.253		
118	3-Phenylpropyl isothiocyanate	111 ± 14	0.499		
119	Physcion	132 ± 16	0.180		
120	(1R)-(+)-a-Pinene	88 ± 12	0.567		
121	(1S)-(-)-a-Pinene	111 ± 9	0.537		
122	(1S)-(-)-β-Pinene	109 ± 5	0.202		
123	Plumbagin from Plumbago indica	132 ± 5	0.210		
124	Protocatechuic Acid	152 ± 13	0.106		
125	Quassin	116 ± 15	0.484		
126	Quercetin, Dihydrate	166 ± 15	0.033		*
127	Rebaudioside A	105 ± 4	0.525		
128	Resveratrol	273 ± 60	0.048		*
129	Retinoic acid	109 ± 9	0.708		
130	Rhein	133 ± 37	0.481		
131	Rosmarinic acid	105 ± 17	0.857		
132	Rutin	111 ± 10	0.355		
133	Rutin trihydrate	104 ± 13	0.871		
134	Salicylic Acid Methylester	119 ± 29	0.560		
135	Sarsasapogenin	125 ± 20	0.447		
136	Schaftoside	93 ± 11	0.558		
137	Scopoletin	111 ± 17	0.722		
138	Scutellarein	131 ± 15	0.185		
139	Sennoside	118 ± 14	0.418		
140	Sesamol	117 ± 16	0.432		
141	Shikalkin	99 ± 6	0.865		
142	Shikonin	118 ± 8	0.149		
143	Silibinin	125 ± 11	0.341		
144	Sinomenine	136 ± 11	0.116		
145	Sophocarpine	129 ± 21	0.310		
146	β-Carotene	104 ± 6	0.653		
147	β-Sitosterol	122 ± 1	0.478		
148	Stevioside	112 ± 18	0.591		
149	Swertiamarin	103 ± 5	0.634		
150	Tannin < Tannic Acid>	145 ± 12	0.072		
151	Tanshinone I	82 ± 9	0.469		
152	Tanshinone IIA	130 ± 7	0.240		
153	(±)-Taxifolin	136 ± 37	0.440		
154	(+/−)-Taxifolin hydrate	110 ± 7	0.680		
155	Terpinyl acetate	103 ± 8	0.844		
156	trans-Ferulic acid	50 ± 5	0.001		**
157	trans-Oxyresveratrol	155 ± 13	0.019		*
158	trans-Piceatannol	205 ± 5	0.0002		***
159	trans-Polydatin (trans-Piceid)	109 ± 12	0.538		
160	trans-Pterostilbene	148 ± 14	0.031		*
161	(+)-trans Taxifolin	128 ± 2	0.271		
162	Trimethylapigenin	154 ± 25	0.106		
163	Ursolic acid	100 ± 5	0.994		
164	Vanillic Acid	105 ± 5	0.516		
165	Xanthophyll <Lutein>	110 ± 5	0.281		
166	Yohimbine hydrochloride	110 ± 14	0.723		

***P < 0.001, **P < 0.01, *P < 0.05: vs vehicle.

## 2 Experimental design, materials and methods

### 2.1 Screening compounds that increase GAL4-PGC-1β activity

HEK293T cells (Riken Cell Bank, Tsukuba, Japan) were maintained in Dulbecco's Modified Eagle's Medium (DMEM) supplemented with 10% fetal bovine serum (FBS). We used amino acids 1–147 of GAL4 that were fused to the full length of PGC-1β cDNA [2]. Namely, full-length PGC-1β cDNA was cloned into the pM vector (Clontech/Takara Bio, Shiga, Japan) to produce a fusion protein with the GAL4 DNA-binding domain. HEK293T cells were co-transfected with a reporter gene containing four copies of a GAL4 binding site ((UAS)4-Luc), and pM- PGC-1β (GAL4- PGC-1β). The luciferase reporter plasmid (25 ng), expression plasmid (pM- PGC-1β: 25 ng), and the phRL-TK vector (2 ng: Promega Co., Madison, WI, USA) as an internal control of transfection efficiency were transfected into HEK293T cells using Lipofectamine 2000 (Invitrogen, Carlsbad, CA, USA). Five hours after transfection, the cells were plated at a density of 1 × 10^5^ cells per well in a 96-well plate in Dulbecco's modified Eagle's medium (DMEM) supplemented with 10% fetal bovine serum (FBS). Twenty-nine hours after transfection, the cells were treated with various commercially available compounds (Sigma-Aldrich Japan, Tokyo, Japan; final concentration, 10 μM). After twenty hours, cells were lysed and assayed for luciferase activity using the Dual-Glo Luciferase Assay kit (Promega). The activity was calculated as the ratio of firefly luciferase activity to Renilla luciferase activity (internal control) and expressed as an average of triplicate experiments. Namely, the firefly luciferase value was divided by the corresponding Renilla luciferase value. The luciferase values in the presence of vehicle alone were set at 100. The relative values in the presence of indicated compounds are shown.

### 2.2 Statistical analyses

Statistical analyses were performed using the Student's two-tailed unpaired t-test. P value < 0.05 was considered significant.

## References

[1] Uchitomi R., Nakai S., Matsuda R., Onishi T., Miura S., Hatazawa Y., Kamei Y. (2019). Genistein, daidzein, and resveratrols stimulate PGC-1β-mediated gene expression. Biochem. Biophys. Rep..

[2] Kamei Y., Ohizumi H., Fujitani Y., Nemoto T., Tanaka T., Takahashi N., Kawada T., Miyoshi M., Ezaki O., Kakizuka A. (2003). PPARgamma coactivator 1beta/ERR ligand 1 is an ERR protein ligand, whose expression induces a high-energy expenditure and antagonizes obesity. Proc. Natl. Acad. Sci. U.S.A..

